# Extract from Dr. O’Beirne’s Observations on the Nature and Treatment of Dropsy

**Published:** 1842-12-17

**Authors:** 


***Extract from Dr. O'Beirne's Observations on the Nature and Treatment of Dropsy.***—It appears, then, that the general results of my inquiries on the subject of dropsy, are

First, that all the phenomena of the disease are but the products of venous obstruction; and that venous obstruction is caused either by diminished capacity of the lungs, or by an increase of the circulating mass of venous blood, or by both of these causes combined.

Secondly, that the disease is not of an inflammatory nature.

Thirdly, that the disease, with the exception of the early part of its course, is attended with more or less of general debility.

With these general results before me, let me now see what curative indications respecting hydrothorax and anasarca, can be deduced from them. The first of these results manifestly shows, that we have only to remove the venous obstruction, in order to remove the disease; and, that this must be done either by increasing the capacity of the lungs, or by diminishing the circulating mass of venous blood.

With respect to the first of these alternatives, the capacity of the lungs is, in dropsy, either natural or diminished. When natural, as in cases arising solely from either cold or plethora, it would be absurd even to imagine that it could be increased by any means; and when diminished, as in cases arising from hepatization and a variety of other causes, it cannot be increased without removing those causes, and consequently, without losing time which cannot be spared. It appears therefore, that we must fall back upon and adopt the other alternative, that of diminishing the circulating mass of venous blood. This being, then, the main curative indication, how is it to be carried into effect? Seeing that our object should be to relieve the obstructed state of the venous system as fully and as quickly as possible, it is clear that we should employ venesection in preference to cupping, which acts less energetically and more slowly. But what quantity of blood should be taken? The second of the general results shows, that we have not to contend with an inflammatory disease, and consequently that the quantity should not be large; while the third of these results warns us against taking more than a moderate, or rather a small quantity, perhaps from eight to ten ounces. Should we repeat the bleeding? One of the immediate effects of the first bleeding should be that of relieving the absorbent system, and enabling it to restore the effused serum to the circulating mass of blood; and we see a proof of this being actually the case in the evident reduction which venesection causes, even in a few hours, in the swelling and œdema of the external parts. It may be safely inferred, therefore, that a short time is sufficient to restore to the venous system as much serum as compensates for, if it does not exceed, the quantity of blood which had previously been taken from the arm. We see the proof of this, also, in a fact, which I have often observed, that although the patient may at first, bear the bleeding badly, he evidently rallies in a few hours. If necessary, therefore, we need not feel any dread of repeating the bleeding. How often, at what intervals, and in what quantity should it be repeated? It should be repeated when absorption and the secretion of urine become languid, but it will rarely be required to do so more than three or four times during the treatment; the intervals between each should be from two to three or four days, so as not to induce debility; and, for the same reason, the quantity of blood taken should be reduced in succession from eight or ten to six, and from six to four ounces, the last being the smallest that can be of any decided service. If the patient be young, but of weakly constitution, or if he be old and feeble, and supposing, at the same time, that the disease has arrived at a considerable height, how are we to proceed? We should bleed him from a small orifice, and as much as possible in the incumbent posture; while the blood is flowing, we should give him gin and water in such quantities as may be necessary to support him; and, after the arm has been tied up, he should be ordered to have frequently during the day strong broths, and such other kinds of animal food as may be found to agree with him. This mode of treatment will, no doubt, appear very strange, yet it is founded upon the soundest principles, and I have employed it, as will be seen, with the most decided success. The proportions in which I have been in the habit of directing gin and water to be used, are one part of the former to four or five parts of the latter, and the gin should be Dutch, not English. It is scarcely necessary to say, that I have selected this kind of spirituous liquor on account of its well-known diuretic properties. If a case be so far advanced that the cavity of the pleura is filled, or nearly so, with serous fluid, and if the patient be evidently dying from difficulty of breathing, yet not comatose or paralysed, are there any means of immediately relieving the difficulty of breathing, so as to enable us to give him a chance for his life? I cannot answer this question from experience, but it appears to me to be a case, particularly if the patient be young and has been previously healthy, in which we would be justified in instantly having recourse to paracentesis, which may be performed by a common lancet, if we should at the time not happen to have a better instrument. While the fluid is issuing from the chest, the patient should be given warm wine and water, and every restorative means should be employed; and, when a quantity sufficient to relieve his breathing has been drawn off, the wound should then be carefully closed with adhesive straps. This being done, and the restorative plan being continued, it is probable that absorption will go on, and enable us in a few hours, to find a vein from which we should take blood in a quantity proportioned to the patient's strength, from a small orifice, and in the recumbent posture. Having thus reduced the case to the state of an ordinary one, we may then treat it as such, until, perhaps, success shall have, at length, crowned our efforts to snatch a victim from the very mouth of the grave.

The next curative indication is obviously to support the patient by animal food, and the occasional use of gin and water, in order to enable him to bear not only these repeated bleedings, but also the debilitating effects of the disease. The only exception to this rule is, perhaps, the case of a young strong person, in whom an attack of inflammation of the lungs has just terminated in serous effusion. In such a case, it will be advisable to withhold both animal food and spirituous drinks for two or three days, but beyond this time, if not before, the state of the patient will not, even in this case, fail to evince the necessity of giving both.

The other curative indications are, first, to free the bowels, if confined, with compound powder of jalap, or, if their secretions and discharges be unhealthy, with calomel or blue pill; secondly, to assist the action of the kidneys by diuretics; thirdly, if the disease has arisen from hepatization of the lung or lungs, to administsr blue pill in combination with diuretic powders, as this combination often acts upon the mouth as well as the kidneys, and, by doing so, causes absorption of the coagulable lymph deposited in the cellular tissue of the lungs; fourthly, should more or less of hepatization remain after the dropsy has been removed, to employ a mild mercurial course until all dulness has disappeared, and the natural respiration is again heard all over the chest; fifthly, when the disease has been completely removed, to advise generous diet, tonics, and change of air.

Such is the treatment which I recommend in hydrothorax, and also with a few obvious modifications in anasarca, and I shall now proceed to show that it is one which I have successfully employed for nearly twenty years.

I should now proceed to detail cases of anasarca successfully treated by the same means, but as that disease is really but a mild form of hydrothorax, and as almost all the cases of it that I have met, yielded readily to a single bleeding, with diuretics and purgatives, I consider that the detail of such cases would but uselessly occupy the time of the reader. But I shall take the liberty of directing his attention to some facts respecting the use of venesection in dropsy, which may enable him to see at once the precise difference between my treatment of the disease, and that hitherto recommended and employed. Venesection is recommended by Hippocrates, and many ancient and modern authors; and about the middle of the sixth century, Alexander Tralles, or Trallian, advocated it *per exiguas missiones*, and his successors, down to Maclean, Blackall, and still later writers on the subject, have adopted the improvement. There is nothing new, therefore, in the treatment of the disease by small and repeated bleedings. But it will be found that, without any exception, all of them have restricted venesection by so many conditions respecting the age, strength, and temperament of the patient; the organ affected; the state of the pulse and respiration; the urine being or not being albuminous; the origin of the disease from inflammation, or from the suppression of either natural or accustomed discharges; the occurrence of hæmoptysis; and a great variety of other points, that the use of this valuable remedial means really constituted the exception, not the rule. In fact, it was, and now is, so very conditional, that many have been deterred from having recourse to it in any case. Again, even the modern works on the subject exhibit the clearest admissions of the great difficulty to be encountered in steering a safe course between, on the one hand, the obvious necessity for counteracting the tendency to debility, and, on the other, the danger of exasperating a diathesis supposed to be inflammatory. But, now that the practitioner has before him views which avoid the extreme of both doctrines; now that he is supplied with a set of clear and simple rules to direct him in bleeding; now that he sees and must be convinced that he can not only support, but stimulate the patient with the greatest advantage, and without any risk; it is to be hoped that he will, sooner or later, surrender his prejudices against the use of the lancet, and adopt a practice which will not, I venture to assert, fail to answer all his reasonable expectations.—*Ibid.*

